# Temperature-dependent infrared ellipsometry of Mo-doped VO_2_ thin films across the insulator to metal transition

**DOI:** 10.1038/s41598-020-65279-4

**Published:** 2020-05-22

**Authors:** S. Amador-Alvarado, J. M. Flores-Camacho, A. Solís-Zamudio, R. Castro-García, J. S. Pérez-Huerta, E. Antúnez-Cerón, J. Ortega-Gallegos, J. Madrigal-Melchor, V. Agarwal, D. Ariza-Flores

**Affiliations:** 1 Instituto de Investigación en Comunicación Óptica, Universidad Autónoma de San Luis Potosí, Alvaro Obregón 64, San Luis Potosí, S.L.P. 78000 Mexico; 2 CONACYT-UASLP, Instituto de Investigación en Comunicación Óptica, Universidad Autónoma de San Luis Potosí, Alvaro Obregón 64, San Luis Potosí, S.L.P. 78000 Mexico; 3 Unidad Académica de Ciencia y Tecnología de la Luz y la Materia, Universidad Autónoma de Zacatecas, Carretera Zacatecas-Guadalajara km. 6, ejido la Escondida Campus UAZ Siglo XXI edificio E8, Zacatecas, Zac. 98160 Mexico; 4 Centro de Investigación en Ingeniería y Ciencias Aplicadas, Universidad Autónoma del Estado de Morelos, Av. Universidad 1001 Col. Chamilpa, Cuernavaca, Morelos 62210 Mexico; 5 Drug Delivery, Disposition & Dynamics, Monash Institute of Pharmaceutical Sciences, Monash University, Parkville, VIC 3052 Australia; 6 Melbourne Centre for Nanofabrication, Victorian Node of the Australian National Fabrication Facility, Clayton, VIC 3168 Australia

**Keywords:** Optics and photonics, Applied optics, Optical materials and structures, Optical techniques

## Abstract

We present a spectroscopic ellipsometry study of Mo-doped VO_2_ thin films deposited on silicon substrates for the mid-infrared range. The dielectric functions and conductivity were extracted from analytical fittings of Ψ and Δ ellipsometric angles showing a strong dependence on the dopant concentration and the temperature. Insulator-to-metal transition (IMT) temperature is found to decrease linearly with increasing doping level. A correction to the classical Drude model (termed Drude-Smith) has been shown to provide excellent fits to the experimental measurements of dielectric constants of doped/undoped films and the extracted parameters offer an adequate explanation for the IMT based on the carriers backscattering across the percolation transition. The smoother IMT observed in the hysteresis loops as the doping concentration is increased, is explained by charge density accumulation, which we quantify through the integral of optical conductivity. In addition, we describe the physics behind a localized Fano resonance that has not yet been demonstrated and explained in the literature for doped/undoped VO_2_ films.

## Introduction

Vanadium dioxide (VO_2_) undergoes a first-order insulator-to-metal transition (IMT) at a critical temperature (${T}_{c}$) around 68 °C^1,2^, which is accompanied by a structural transformation of a low-temperature monoclinic (M, insulator) to high-temperature tetragonal rutile (R, metal) phase. This transition is fully reversible and is a result of an interplay between Mott and Peierls mechanisms^3,4^, whereas the relative contributions are still a subject of debate^5^. The monoclinic to rutile or intermediate monoclinic phase transitions coincide with large changes in the optical^6^, electrical^7^, thermal^8^, and magnetic properties^9^, making VO_2_ one of the most intensively studied phase change materials due to its unique temperature-dependent physicochemical properties and, at the same time, opens exciting new opportunities for the development of novel functional materials with a wide range of technological and industrial applications such as radiative thermal memristors^10^, ultrafast tunable nanoplasmonics^11,12^, uncooled bolometers^13^, energy-efficient smart windows^14–17^, heat storage devices^18^, temperature sensors^19^, ultrafast tunable opto-electronic nano-gating^20^, room temperature hydrogen nanosensors^21^, infrared optical limiters^22^, memory and neuromorphic devices^4^, and tunable terahertz metamaterials^23^, among others.

Some of the above applications critically rely on the performance of the VO_2_ IMT close to ambient conditions. To enhance the application of VO_2_ in thermally-triggered optical and electrical switching devices, for instance, the shifting and effective control of its phase transition temperature to around room temperature is highly desirable. Faced with this challenge, it is important to highlight that the critical temperature at which VO_2_ phase transition occurs, is substantially affected by the degree of impurities embedded in the bulk of the material as dopants which, for a few percent concentration can induce an appreciable change of ${T}_{c}$. For example, a conventional approach to regulate the VO_2_ IMT is controlling the carrier concentration in the material, that is, by selectively choosing the type and level of dopant element, an electron- or hole-injection can be built up into the bulk material. Thus, doping the VO_2_ lattice with high-valence metal transition ions such as W^6+^, Mo^6+^, or Nb^5+^ effectively decreases the ${T}_{c}$, whereas low-valence metal ions such as Cr^3+^, Al^3+^, Fe^3+^, or Ga^3+^ are found to severely increase it^24–29^.

Moreover, the fabrication of VO_2_ crystalline thin films has been carried out by different techniques such as pulsed laser deposition^30–32^, sputtering^16,33^, molecular beam epitaxy^34^, dip-coating^35^, chemical vapor deposition^36^, spin coating^19^, etc. In particular, the fabrication of VO_2_ thin films by spin coating deposition of a sol-gel precursor is one of the easiest and low cost techniques to produce reasonably high quality films, with an additional advantage of not requiring sophisticated equipment. The latter opens the possibility to scale up the fabrication process of VO_2_ coatings enabling, thus, the practical application in industrial fields by developing an efficient and straightforward synthesis method^19^. Moreover, it has been demonstrated that only minor discrepancies appear in the optical properties of samples fabricated by spin coating deposition as compared to the samples prepared with the sputtering technique^37^.

On the other hand, the strongly correlated conducting state of VO_2_ has been associated to nanoscale metallic puddles that percolate at the IMT^38^. Near-field microscopy observations along with infrared spectroscopic ellipsometry (IRSE), show that the coalescence of metallic puddles are directly related to the significant increment of the optical conductivity, and the divergence of the real part of the dielectric function in the low frequency limit, when the percolation is taking place^39^. In parallel to these findings, Walther *et al*.^40^, interpret the terahertz conductivity of ultrathin metallic gold films at the percolation transition in terms of the carrier backscattering Drude-Smith model^41^. The extracted parameters of the Drude-Smith model, namely, the plasmon frequency ${\omega }_{p}$, collision-modified lifetime, $\tau$, and persistence of original velocity after collisions $c$, provide a reasonable explanation for the percolative behavior of gold thin films at the IMT, as compared to the unflexible Drude model^40,41^. Consequently, the Drude-Smith model can be applied to describe temperature-dependent optical ellipsometry measurements in order to elucidate the electronic correlations around the percolation threshold of Mo-doped VO_2_ thin films.

As an optical technique, spectroscopic ellipsometry has proved to be a non-invasive and non-destructive powerful probe for monitoring the optical properties of VO_2_ thin films through IMT, e.g. Kana *et al*., studied the thermally tunable optical constants of VO_2_ thin films in the visible to near infrared (NIR) range^42^. Lafort *et al*., determined the refractive indices of sputtered films of VO_2_ deposited on stainless steel substrates^33^. Huang *et al*., carried out an analysis of the phase transition of VO_2_ thin films by combining Raman scattering and ellipsometry for visible and NIR range^43^. More recently, Wan *et al*., reported the influence of substrate and fabrication method on the complex refractive index of VO_2_, along with Looyenga mixing rule fittings at the IMT, in oder to describe the coexistence of insulating and metallic domains as an effective medium^37^. Furthermore, the Bruggeman effective medium theory, successfully used to obtain the response of inclusions in media, has been reported to fail in the regime near the percolation transition (for the mid-IR range^44^) and also has been inconsistent with optical measurements in the far IR range^40^. While effective medium theories are useful to describe the dielectric function of mixtures, they do not explain some fundamental phenomena that occur in the microstructure of VO_2_.

Although some studies of IRSE have been carried out in vanadium oxide^33,37,42–46^, to the best of our knowledge, the influence of dopants on the optical properties of VO_2_ through the IMT has not yet been analyzed by Drude-Smith model. In this article, we study the temperature-dependent mid-infrared optical properties of Mo-doped VO_2_ thin films across the IMT using the spectroscopic ellipsometry technique. From our measurements, we are able to determine the complex dielectric functions and optical conductivities of Mo-doped samples throughout the thermally induced transition. We show that the parameters, as extracted from the Drude-Smith model and the changes of the phase of a Fano resonance, provide a consistent description of the most pronounced infrared optical effects carried out at the percolation transition of Mo-doped and undoped VO_2_ layers.

## Materials and Methods

### Synthesis of Mo-doped VO_2_ thin films

All the reagents were of analytical grade and used as obtained commercially without further purification. Vanadium pentoxide (V_2_O_5_) was selected as the starting material, oxalic acid (H_2_C_2_O_4_ · 2H_2_O) as the reducing agent and ethanol as the solvent. Ammonium molybdate tetrahydrate ((NH_4_)_6_Mo_7_O_24_ · 4H_2_O) was used as the dopant supplier substance to decrease ${T}_{c}$. Substrates were prepared from (001)-oriented, boron doped p-type silicon wafer (1–5 mΩ · cm). Piranha solution was used for cleaning and pre-treating the silicon surface.

VO_2_ thin films were prepared using a chemical-based fabrication method^19,47^. Briefly, vanadium pentoxide V_2_O_5_ and H_2_C_2_O_4_ · 2H_2_O were mixed in ethanol in a molar ratio of 1:3, respectively. A transparent blue vanadyl oxalate (VOC_2_O_4_ · *x*H_2_O) precursor solution was obtained after refluxing at 120 °C for 10 h, followed by the filtration of the precursor (to remove any unreacted chemicals) and storage under an inert atmosphere to avoid further oxidation. Substrates were immersed for 24 h into a solution of H_2_SO_4_:H_2_O_2_ in a volumetric ratio of 3:1, in order to remove residual organic contamination and improve the hydrophilicity of the silicon surface (hazards of working with piranha solution can be found in ref. ^48^). Mo-doped VO_2_ thin films were synthesized by the addition of (NH_4_)_6_Mo_7_O_24_ · 4H_2_O (initially dissolved in few drops of deionized water) to achieve the dopant levels (1% and 2% with respect to vanadium) and then, slowly added to the VOC_2_O_4_ · *x*H_2_O precursor under vigorous stirring followed by ultra-sonication during 2.5 h to obtain a homogeneous precursor solution. Along with the control sample (0% dopant), Mo-doped VO_2_ thin films were deposited by spin coating the precursor solution at 3000 rpm for 30 s over the pre-treated silicon substrate in a nitrogen atmosphere. After drying at 100 °C to remove the excess solvent, the prepared samples were thermally annealed in a tube furnace at 450 °C for 60 min at a heating rate of 10 °C/min in nitrogen atmosphere to form a crystalline VO_2_ thin film. The morphological properties and thickness of the films were observed through field emission scanning electron microscopy (SEM, Hitachi SU5000).

### Optical characterization

A commercial variable-angle infrared spectroscopic ellipsometer IR-VASE from J.A. Woollam Co. Inc, equipped with a Peltier-based homemade temperature control system, was used to characterize the optical properties of VO_2_ thin films as a function of temperature. The ellipsometer consists of an infrared source (BOMEM MB102), a positionable polarizer, a high precision $\theta -2\theta$ sample stage, a rotating compensator, and an analyzer, with computerized controls. This configuration is known as PSC_R_A^49^. A paraboloidal mirror (with a 50 cm focal length) focuses the beam to an 8 mm spot diameter on the surface of the sample. Spectral intensity is acquired with a DTGS detector. The signal is then automatically reduced to the ellipsometric angles Ψ and Δ, defined below, as functions of frequency from 300 to 6000 cm^−1^ (1.5–33 *μ*m). For the experiment, we set an angle of incidence of 67° with a nominal spectral resolution of 16 cm^−1^. These experimental settings were considered sufficient to permit synchronization along with the temperature control with reasonable scanning and thermal stabilization times. Furthermore, the temperature control was implemented to work in setting-only mode, i.e., without feedback cycles, such as e.g., PID, which would have induced undesired hysteresis sub-loops within the main loop, that would have distorted the final results. On the other hand, due to the heavily doped substrates used in the present study, the absorption contribution of free-charge carriers impedes the IR radiation to reach the backside of the samples; thus no backside surface treatment was required for our samples as it is needed for intrinsic silicon to avoid spurious reflections^37^.

Ellipsometry measures the changes in the polarization state of linearly polarized incident light upon reflection off the sample surface. This change is expressed as the ratio of the complex Fresnel reflection coefficients ${r}_{p}$ and ${r}_{s}$ for light polarized parallel and perpendicular to the plane of incidence, that is to say^49,50^

1 $$\rho =\frac{{r}_{p}}{{r}_{s}}=\,\tan (\Psi ){e}^{i\Delta }$$

where tan(Ψ) is the ratio of the magnitudes of ${r}_{p}$ and ${r}_{s}$ and Δ is the relative phase difference between those coefficients. The measured ellipsometric spectra, expressed in terms of Ψ and Δ values as a function of the frequency, are then fitted with the aid of commercial software to obtain the temperature-dependent optical parameters.

## Results and Discussion

**Figure 1:**
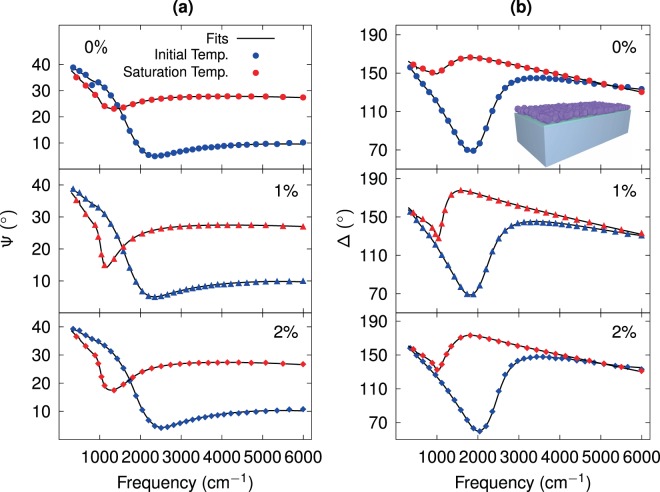
Experimental (circles/triangles/diamonds) and modeled (continuous lines) spectra of (**a**) Ψ and (**b**) Δ ellipsometric angles for Mo doped VO_2_ thin films. Initial/saturation temperature is plotted in blue/red. Corresponding doping percentages are indicated in each plot. The inset illustrates the granular VO_2_ film over the silicon substrate (gray) with a native silicon oxide layer (light blue), the image was created with uMake software, Education Edition, https://www.umake.xyz/.

Figure 1 shows the ellipsometric Ψ (Fig. 1(a)) and Δ (Fig. 1(b)) spectra corresponding to Mo-doped VO_2_ films for three doping percentages from 0 to 2%. The spectra are displayed for initial (blue circles/triangles/diamonds) and saturation temperatures (red circles/triangles/diamonds), along with fittings of experimental data performed through a sum of oscillators (black solid lines), as discussed in the latter part of this paper. The fits were obtained by employing the CompleteEASE ellipsometry software from J.A. Woollam, considering a multilayered system composed of the VO_2_ layer, a native silicon oxide layer, and a semi-infinite *p*-type $\mathrm{(001)}$ silicon substrate (schematically shown in the inset of Fig. 1(b)). A good agreement between experimental spectra and modeled line shapes can be observed. Additionally, temperature-dependent experimental runs and spectra calculations for the bare SiO_2_/Si substrate were performed separately in order to leave the dielectric functions and thicknesses corresponding to VO_2_ films as unique goals of the fitting procedure. The thicknesses of VO_2_ films and native silicon oxide layer, determined from spectroscopic ellipsometry measurements (*d*_IRSE_ and *d*_Ox_, respectively) and scanning electron microscopy (*d*_SEM_), are shown in Table 1. The difference in the thicknesses determined by ellipsometry from those obtained by SEM, can be attributed to the following factors: (i) the area of the light spot of the ellipsometer of ~1 cm^2^, being much larger than the SEM observation zone (<400 nm) leads, thus, to an average measurement with different thicknesses over the sample; (ii) the modeled dielectric function can be the result of a non-uniform mixing of different dielectric functions of different regions of the film, whereby an effective dielectric function of the film can produce a different optical thickness, resulting in a mismatch of optical and SEM widths; and (iii) the surface roughness of the film is not considered in this study.

**Table 1:** Thicknesses of VO_2_ and native silicon oxide layers, determined from IRSE (*d*_IRSE_ and *d*_Ox_), and SEM (*d*_SEM_).

Mo dopant (%)	*d*_IRSE_ (nm)	*d*_SEM_ (nm)	*d*_Ox_ (nm)
0	56.2 ± 0.1	69 ± 10	8.1 ± 0.1
1	58.4 ± 0.1	53 ± 10	8.1 ± 0.1
2	55.8 ± 0.1	62 ± 9	8.1 ± 0.1

**Figure 2:**
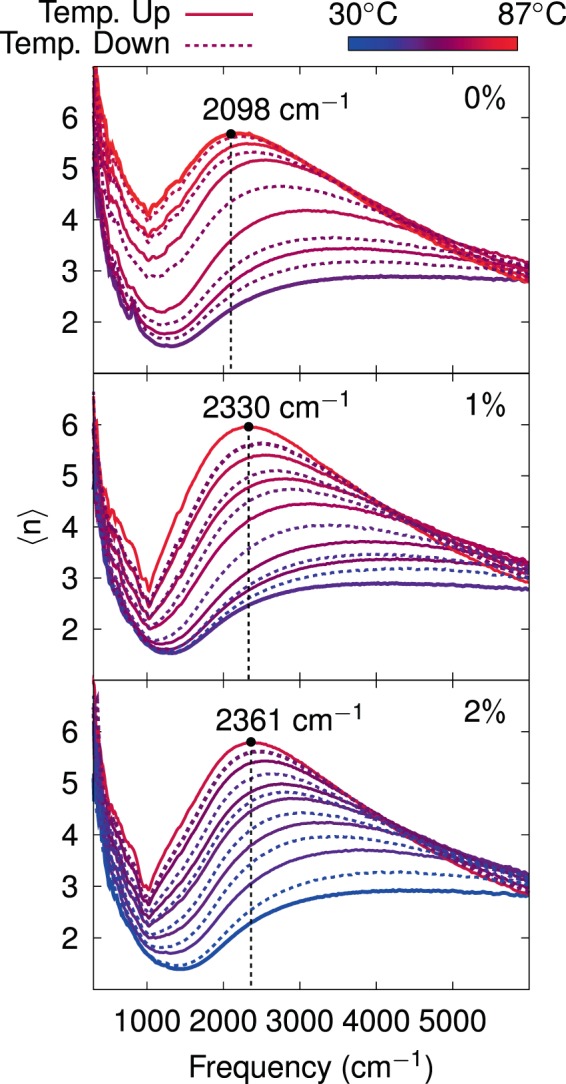
Representative spectra of pseudo-refractive index as a function of the frequency for different temperatures across the IMT. Solid and dashed lines correspond to rising and lowering temperatures, respectively. Black circles indicate the maximum value of pesudo-refractive index in the saturation temperature curve. Corresponding doping percentages are indicated in each plot.

Figure 2 shows selected experimental spectra of the pseudo-refractive index $\langle n\rangle$ (also termed pseudo-transform) of Mo-doped VO_2_ thin films for the different doping percentages. The plots show the evolution of $\langle n\rangle$ from initial lower temperature, (chosen to be close the IMT), gradually increasing and changing its spectral line shape up to finally reaching an unchanging state at a particular saturation temperature (solid lines). To complete the measurement cycle by decreasing temperature, the $\langle n\rangle$ spectra gradually decrease to reach again the initial state (dashed lines). The symbol $\langle n\rangle$ corresponds to the effective refractive index obtained from inversion of Ψ and Δ angles, using an expression that provides reliable dielectric functions only for bulk-like samples (see Eq. 1 in the Supplementary Information). In the case of stacked media, $\langle n\rangle$ has contributions from the complete layered structure schematically shown in the inset of Fig. 1(b), i.e., it is a convolution of optical properties and thicknesses of individual layers that must be revealed through a model. The justification for showing these pseudo transforms is rooted in a couple of practical reasons. First, among the available representations of ellipsometric data, $\langle n\rangle$ spectra provided the best way to monitor changes during experimental thermal runs, due to their qualities of being always positive and growing nearly monotonically for the present system throughout the complete working spectral range, and second, the line shape of this representation is found to have a nice resemblance (except for the broad dip at ~1500 cm^−1^ produced by the substrate), to the optical conductivity extracted from the model (the results of optical conductivity and a further justification for using $\langle n\rangle$ are presented below). For the sake of clarity, we only show the real part of the pseudo-refractive index, whereas the imaginary part $\langle k\rangle$ and pseudo-dielectric transforms are shown in the Supplementary Information (Figs. S1 and S2). As the temperature is increased, the $\langle n\rangle$ spectrum rise with a marked hump (observed between 2000 and 2400 cm^−1^, depending on dopant concentration) suggesting collision-rich percolative conductivity, which can be modelled by the Drude-Smith characteristic line shape^41^. Furthermore, the dip at around 1000 cm^−1^ which appears to be more pronounced as the conductivity is increased, implies the presence of an asymmetric Fano-like resonance^51^. The variation of the refractive index of Si substrate, as a function of the temperature, is negligible as compared to the change of VO_2_ layer (see Fig. S3 of Supplementary Information), therefore, the huge variation of $\langle n\rangle$ has been mainly attributed to the contribution of thermochromic changes in VO_2_, which are also affected by the doping level. For VO_2_ films, a series of phonon resonances are present in the region below 1000 cm^−1^ ^52^, however, in this work phonon effects (although important for the determination of the crystallographic phase), are not considered in the extraction of the dielectric function, since we are interested in the very broad conductivity spectral contribution. Moreover, none of the reported vibrational modes coincides with our so-assigned Fano-like resonance. In fact, with the sharpening of the Fano structure, the phonon resonances tend to smear out.

**Figure 3:**
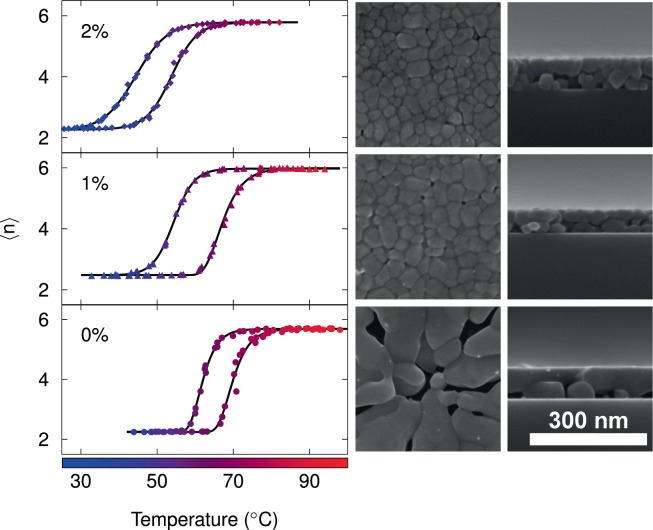
Hysteresis loops collected at a frequency of the maximum value of $\langle n\rangle$ for the saturation temperature. Experimental measurements are plotted in circles, triangles and diamonds for 0%, 1% and 2%, respectively; solid lines correspond to fits via either Gompertz or logistic growth laws. Each loop is composed of 3 experimental runs. Right panels show a set of SEM images of the samples surface (left column) and the cross sections (right column).

Figure 3 shows the hysteresis loops for different doping levels, constructed from the pseudo-refractive index functions, by extracting data of traces corresponding to the frequency where the maximum value of $\langle n\rangle$ is located at saturation temperature (vertical dashed lines in Fig. 2). The experimental data, i.e., each individual symbol in Fig. 3, corresponds to an average taken over eleven neighboring spectral points around the trace frequency. The selection of $\langle n\rangle$_max_ as a representative of experimental spectra as a function of the temperature over other visualizations of ellipsometric data demands a justification besides its resemblance to optical conductivity: it is also more sensitive to temperature-driven changes even from the onset of the IMT as compared to hystereses of e.g., $\langle {\varepsilon }_{1}\rangle$, which show a delayed response to temperature, at least for the present system. We elaborate on this result in the context of Figs. S4 and S5 of Supplementary Information. The continuous black lines correspond to fitted line shapes, performed separately for increasing and decreasing temperatures, via Gompertz^53^ (characterized by its asymmetric “S”-shape, used here for the ascending temperature of 0% and 1%, and descending temperature of 0% of Mo-doping) and logistic^54^ (of symmetric “S”-shape, used for both, ascending and descending temperatures for the rest of the data) growth laws, which are non-linear models to predict e.g., population growth in confined spaces, or with some other restrictions are widely used in the fields of marketing, insurance, health and biology (for instance for populations of plants^55^ and and bacteria^56^). On comparing different samples, apart from the shift in the hysteresis loops to lower temperatures with increasing doping level, the slopes at the inflection temperature are found to decrease, i.e., a higher amount of Mo-dopant leads to a more gradual IMT (data from the quantitative analysis are presented in the Supplementary Information). Transition temperatures exhibit a linear diminution with increasing Mo concentration. Concerning the morphology, it is observed (see right panels in Fig. 3) that both, the doped and undoped VO_2_ films present uniform coverage of the substrate and a homogeneous, granular texture whose average grain size seems to depend on the percentage of dopant^19^. As the growth conditions for all the samples were kept the same, the observed differences in the morphology of the films have been attributed to the variation in the dopant concentration. Although, the present results are in conformity with some reported observations^46,57,58^, nevertheless we do not discard some slight contributions due to variations associated to thermal gradients (along the furnace during the annealing process) and/or to different thicknesses observed in the layers that in turn could lead to the formation of different crystallization morphologies induced by strain relaxation^37^.

**Figure 4:**
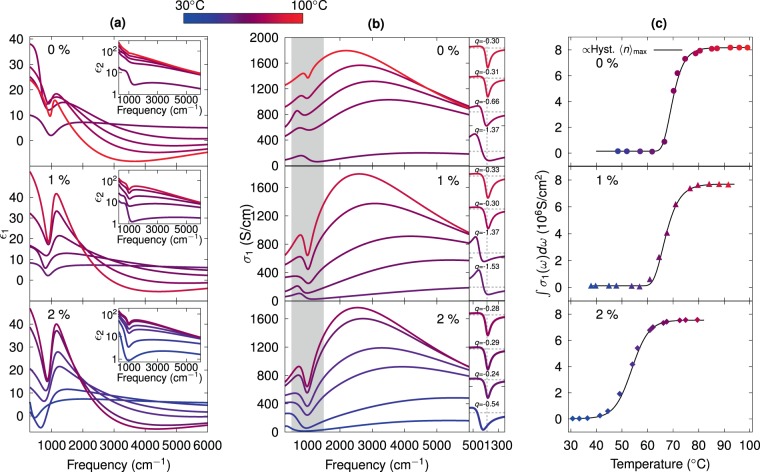
(**a**) Real and imaginary (insets) parts of the dielectric function, and (**b**) real part of the optical conductivity, for a set of selected temperatures across the IMT of Mo-doped VO_2_ thin films. The corresponding doping percentages are indicated in each plot. The color barscale represents the temperature of each measured spectra. The right panels in (**b**) display the Fano line shapes for selected values of temperature for the doping percentage indicated in the left. (**c**) To illustrate the *f*-sum rule, the integrals of conductivity spectra in (**b**) for different temperatures (symbols) are compared to carriers density growth laws reproduced, adjusting scale, from Fig. 3 (solid lines) for the corresponding doping levels.

Figure 4 shows the extracted dielectric function and conductivity spectra of Mo-doped VO_2_ thin films, for different temperatures across the IMT. We note that by dielectric function we are referring to the relative, adimensional one throughout this article. The extraction of the dielectric function was carried out by fitting of experimental data via a sum of oscillators. In the case of VO_2_ layers, we did not consider the granular structure of the films, thus, the extracted dielectric functions correspond to effective media composed of air and VO_2_ grains. To ensure consistency of the modeled optical response across samples with different doping values, the dielectric function was fitted with the same set of oscillators only employing different values for the fitting parameters. For temperatures across the IMT, Fano and Drude-Smith oscillators were employed due to the inverted asymmetric line-shape resonance observed around 1000 cm^−1^ and the strong absorption of the layer in the metallic phase characterized by a broad peak with maximum, depending on Mo concentration, between 2000 and 3000 cm^−1^ (improperly fitted by the classical Drude theory), respectively^41^ (see Fano and Drude-Smith equations along with a selected deconvolved spectrum in Fig. S5 in Supplementary Information). The silicon substrate dielectric function used in the model for fitting all $\langle n\rangle$ was independently adjusted from a reference sample subjected to a temperature cycle as done for the VO_2_ main samples. For its optical model, we employed temperature-dependent Drude oscillators to emulate the overall line shape and a small correction with a Fano resonance, characteristic of heavily *p*-type doped Si^59^, while the native oxide dielectric function was fitted with two (temperature independent) Gaussian oscillators.

Figure 4(a) displays modeled $\tilde{\varepsilon }={\varepsilon }_{1}+i{\varepsilon }_{2}$ spectra for selected temperatures from 30 to 100 °C. For the undoped sample (top panel, 0%), the real part, ${\varepsilon }_{1}$ is observed to abruptly increase as a function of temperature for some fixed frequencies at the lower spectral end, followed by a subsequent decrease while crossing the ${T}_{c}$, in agreement with the first order transition divergence reported by Qazilbash *et al*.^38^, however, we were unable to reproduce the complete behavior due to the lack of data in the low frequency terahertz range. In contrast, for doped samples (middle and bottom panels, 1% and 2% of Mo-doping, respectively) we observe a more gradual transition close to zero frequency, but similar line shapes for the rest of the spectra, with a clear metallic behavior characterized by negative values of ${\varepsilon }_{1}$ for a broad range of frequencies with (nearly) monotonic trend. The imaginary part (${\varepsilon }_{2}$) reveals a gradual increment with increasing temperature for whole spectral range (insets). The real part of the conductivity, in S.I. units, related to the imaginary part of dielectric function via

2 $${\sigma }_{1}(\omega )={\varepsilon }_{0}\omega {\varepsilon }_{2}(\omega ),$$

is shown in Fig. 4(b). For all the samples, an increase in temperature reveals a huge increment of more than two orders of magnitude in the conductivity values, with respect to its value at low temperature. Moreover, the Fano structure (at 1000 cm^−1^) and a clearly Drude-Smith line shape (hump between 2000–3000 cm^−1^) become appreciable with the rise of temperature as metallic phase is reached. Drude-Smith model takes into account carrier localization through enhanced carrier backscattering^41^, and has been reported as an excellent model to describe photoconductivity in InP nanoparticles^60^, ZnO-based nanowires, nanoparticles/thin films^61^, and metal-insulator percolation transitions^40^. Although the Drude-Smith theory has been improved by Cocker *et al*.^62^, we observed excellent fits of our spectra employing the generalization of Smith theory^41^. Additionally, Drude-Smith conductivity displays a maximum value for non-zero frequency, which can not be obtained from Drude model^41^.

Based on reports that the phase of Fano resonance can be modulated for controlled fraction of vacancies between dielectric particles^63,64^, in the present case the origin of the Fano resonance has been attributed to the voids between VO_2_ particles. Here, we can consider that the conducting state contributes to the continuum state and the charges at walls polarizing the voids contribute to the discrete coupling state required by Fano theory^63,64^. In spite of the fact that voids fraction and distances between grains remain constant, the electronic interaction of the walls changes with temperature, resulting in a temperature-dependent Fano response. The right panels of Fig. 4(b), show the shifting $q$ phase of the resonance from negative to near to zero values as the temperature is increased, suggesting an approximation to percolation. In analogy with the report from Pariente *et al*.^63^, we consider the granular VO_2_ structure as quasi-disordered photonic crystals percolating through interactions of polarization charges on its walls, and so interconnecting the different dielectrics. For the partially conducting VO_2_ particles the Fano phase $q\approx 0$ is reached when approaching saturation independently of Mo concentration, which suggests that voids and continuum, although both excited by light are finally decoupled^64^. The variation of parameter *q* as a function of the temperature is shown in Supplementary Information (Fig. S7). This observation is supported by the temperature, morphology and dopant-dependent varying positions of the Fano resonance, whose *q*-phases reach all the same close-to-zero value at saturation and whose line shapes show a pure Lorentz-like, but inverted, resonance independently of doping level and morphology. Furthermore, the larger size of grains and their better interconnection, and the larger sizes of voids for the 0% VO_2_ film (see Fig. 3), may explain the smaller size of its corresponding Fano resonance (reduced wall interactions), its flatter Drude-Smith contribution (more room to extend puddles, slight enhancement in probability of forming connections), and its broader range of negative ${\varepsilon }_{1}$ as compared with the doped (more granular) films. Supporting thus, our interpretation of the origin of the Fano peak in terms of film morphology.

The density of charge carriers, for a given temperature, can be quantified by means of the $f$-sum rule, which states that

3 $${\int }_{0}^{\infty }\,{\sigma }_{1}(\omega ,T)\,d\omega =\frac{{\omega }_{p}^{2}(T)}{8}\propto N(T),$$

where $N(T)$ is the (history-dependent) free charge density at $t$. Equation 3 is valid for both, the classical Drude and the Drude-Smith conductivity approaches (this is shown in the Supplementary Information), indicating that the same charge density is only differently distributed, concerning its response to frequency, forming thus the mid infrared peak^41^. Within the limited spectral range of the present work, we attempted to quantify the fraction of carriers resonating in the mid-infrared range. This can be justified by the presence of the broad bump of ${\sigma }_{1}$ as seen in Fig. 4(b). The integrals of ${\sigma }_{1}$ are presented as symbols in Fig. 4(c), where it can be observed that they also present a trend similar to that shown in Fig. 3. In fact, in Fig. 4(c) we reproduce (scaled) the hysteresis curves already shown in Fig. 3. The match indicates that the above employed growth laws describe quite well, and thus validate their employment to fit each branch of the hysteresis loops, the variations of charge density as a function of temperature: for the undoped film the very slow, nearly imperceptible growth of $N$ at the beginning of the cycle may indicate that there are not enough electrons to screen excitons^65^. As the temperature increases the free charges start to accumulate in spatial puddles producing the very fast rise at the bottom of the hysteresis curve. The interconnection of puddles and the concomitant reduction of space to grow explain the subsequent increment of conductivity and its asymptotic approach to saturation. For the doped samples, the extra electron provided by the Mo atom very likely facilitates a more rapid formation of conducting puddles leading to an initially faster, logistic-like growth (see e.g. 2% Mo film at ~40 °C in Fig. 4(c)).

**Figure 5:**
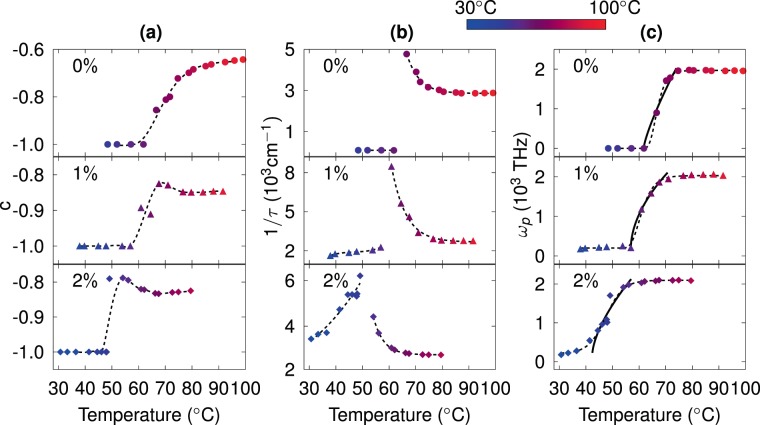
Extracted parameters from Drude-Smith adjustments. (**a**) Persistence velocity, (**b**) inverse of scattering time and (**c**) plasmon frequency for 0% (circles), 1% (triangles) and 2% (diamonds) of Mo-dopant. Dashed lines correspond to smoothed visual guides. Continuous black lines in (**c**) show fitting curves resulting from a power law.

Figure 5 displays the parameters extracted from the adjustments through Drude-Smith model for doped/undoped samples. Figure 5(a) shows the persistence velocity parameter $c$ changing from −1 before percolation transition to −0.65, −0.85, and −0.82 for 0%, 1% and 2%, respectively, in saturation temperature with intermediate values along the IMT range. The negative values of $c$ indicate carrier backscattering, and a clearly non-Drude behavior^41,62^. As the temperature increases, the $c$ value gets closer to zero, indicating a free carrier conduction similar to that of Drude, though never reached. As a comparison, Walther *et al*., reported the change of $c$ parameter for an insulator to metal transition with a clear change from −1 to $0$ in gold films^40^, however, a complete Drude-like behavior ($c=0$) is not observed in our results due to a grain morphology which induces lower values of optical conductivity of VO_2_ in the metallic phase (~10^3^ S/cm) as compared with those of gold (~10^4^–10^5^ S/cm)^40^. Figure 5(b) shows the inverse of scattering time as a function of frequency. Extreme values of ${\tau }^{-1}$ are observed for all doped/undoped samples around the percolation transition. Such divergence indicates a discontinuity of a macroscopic property^40^. For undoped VO_2_, Peterseim *et al*., suggest that while the metallic islands percolate, charges are able to cross the phase borders in the metallic network, but correlations become strong enough to prevent metallic conduction driving the system towards a Mott insulator^6^. We speculate that this divergence can be interpreted as a signature of the first order transition in a Mott semiconductor. Figure 5(c) shows the plasmon frequency parameter changing from 200 to 2000 THz while crossing IMT temperature. The large increment of the plasmon frequency throughout the IMT indicates a more metallic behavior, which is in agreement with the current observations. According to Walther *et al*., plasmon frequency of percolating gold metallic islands can be scaled by ${\omega }_{p}\propto |d-{d}_{c}{|}^{t/2}$, where *d* accounts for the thickness of the layer with a critical value ${d}_{c}$, and $t$ is the critical exponent for a two dimensional system with a value of 1.3^66,67^; SEM observations of gold metallic islands show that the mean diameter proportionally increase with *d*^40^. On the other hand, Qazilbash *et al*., showed near field microscopy direct observations, where the VO_2_ metallic islands grow and coalesce as the temperature increases^38^. Therefore, the mean diameters of VO_2_ metallic islands should be proportional to the temperature, which we can associate, by analogy, to a power law for the plasmon frequency as ${\omega }_{p}\propto |T-{T}_{c}{|}^{t/2}$. Continuous lines in Fig. 5(c) show power fits after percolation threshold with critical exponents of $t/2=0.60$, 0.54 and 0.64 for 0%, 1%, and 2% of Mo-doping, respectively, which are in agreement with reported value of 0.65 for a two dimensional percolative system and supports the electronic correlation assumption for doped and undoped VO_2_ films.

## Conclusions

Mid-infrared spectroscopic ellipsometry measurements were carried out to determine the effective optical constants of Mo-doped VO_2_ thin films, across the IMT. The measured parameters Ψ and Δ angles are fitted by a sum of oscillators as a function of the frequency for different temperatures. For all the doped/undoped films, although unflexible Drude model fails to adjust the experimental data, Drude-Smith model is found to be in good agreement for the complete transition temperature range. While doped samples apparently show a smoother transition as compared to pristine control film, the set of extracted parameters show significant changes involved in the IMT and the discontinuity of a macroscopic property for all doped/undoped films, which are features of a percolation transition. Moreover, the asymmetry of a Fano resonance appearing with increasing temperature, supports the idea of a high electric field emerging in the frontier of the VO_2_ grains due to the percolation in all the proposed (doped/undoped) VO_2_ structures deposited over silicon substrates. The temperature and doping concentration dependent line shapes of thermo-optical hysteresis loops are quantitatively described in terms of growth laws, and are associated to accumulating charge density. As this percolation dependent Fano response is known to be very sensitive to environmental changes and can be tuned with the help of synthesis parameters/temperature, the proposed analysis open new avenues towards a simple and sensitive detection system.

## Supplementary information

Supplementary Information.
