# Supplementary material for: Temperature-dependent infrared ellipsometry of Mo-doped VO2 thin films across the insulator to metal transition
Source: Sci Rep. 2020 May 22;10:8555. doi: 10.1038/s41598-020-65279-4 (PMC7244498; doi:10.1038/s41598-020-65279-4)
Supplement: Supplementary file 1 — Supplementary Information. [file 41598_2020_65279_MOESM1_ESM.pdf]

SCIENTIFIC REPORTS

Supplementary Information for

Temperature-dependent infrared ellipsometry of Mo-doped VO_2 thin films across the insulator to metal transition

S. Amador-Alvarado, J.M. Flores-Camacho, A. Solís-Zamudio, R. Castro-García, J.S. Pérez-Huerta, E. Antunez-Cerón, J. Ortega-Gallegos, J. Madrigal-Melchor, V. Agarwal, and D. Ariza-Flores

david.ariza@cactus.iico.uaslp.mx

This PDF file includes:

Figs. S1 to S7
SI References

Additional optical measurements

Imaginary part of pseudo-refractive index $\langle k \rangle$. Figure S1 shows the imaginary part of the pseudo refractive index for different temperatures across the percolation transition of VO_2:Mo/Si-p++ thin films.

Effective optical responses are calculated from an expression which is valid only for bulk samples, where the inversion of ellipsometric measures quantities really yields the dielectric function (refractive index) of the optical medium under consideration. In the case of layered structures, this inversion is a convolution of optical responses of the different individual layers and their corresponding thicknesses. The inversion relation is given by (1)

$$\langle \tilde{\epsilon} \rangle = \sin^2 \theta_0 \left[1 + \tan^2 \theta_0 \left(\frac{1-\rho}{1+\rho} \right) \right], \qquad [1]$$

where θ_0 is the angle of incidence, and ρ is as defined in Eq. (1) of the main part of the text. Further, the pseudo refractive index $\langle n \rangle + i \langle k \rangle = \sqrt{\langle \tilde{\epsilon} \rangle}$.

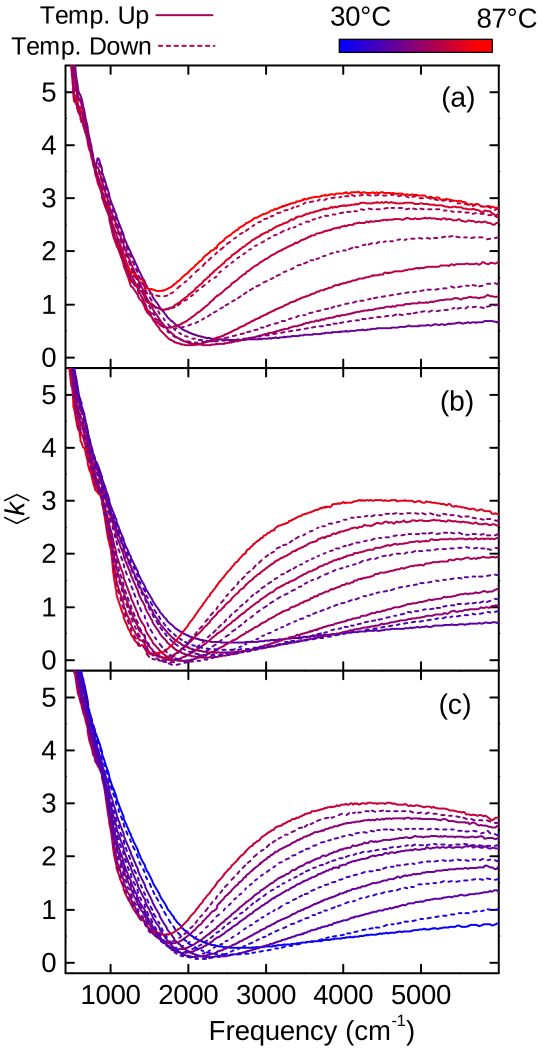

Fig. S1. Representative spectra of the imaginary part of pseudo-refractive index of VO_2:Mo/Si-p++ thin films as a function of the frequency, for different temperatures across the IMT for (a) 0 %, (b) 1 % and (c) 2 %, of Mo-doping. Solid and dashed lines correspond to rising and lowering temperatures, respectively.

Pseudo-dielectric function $\langle\tilde{\epsilon}\rangle$**.** Figure S2 shows the real (left panels) and imaginary (right panels) parts of the pseudo-dielectric function for different temperatures across the percolation transition of VO_2:Mo/Si-p++ thin films.

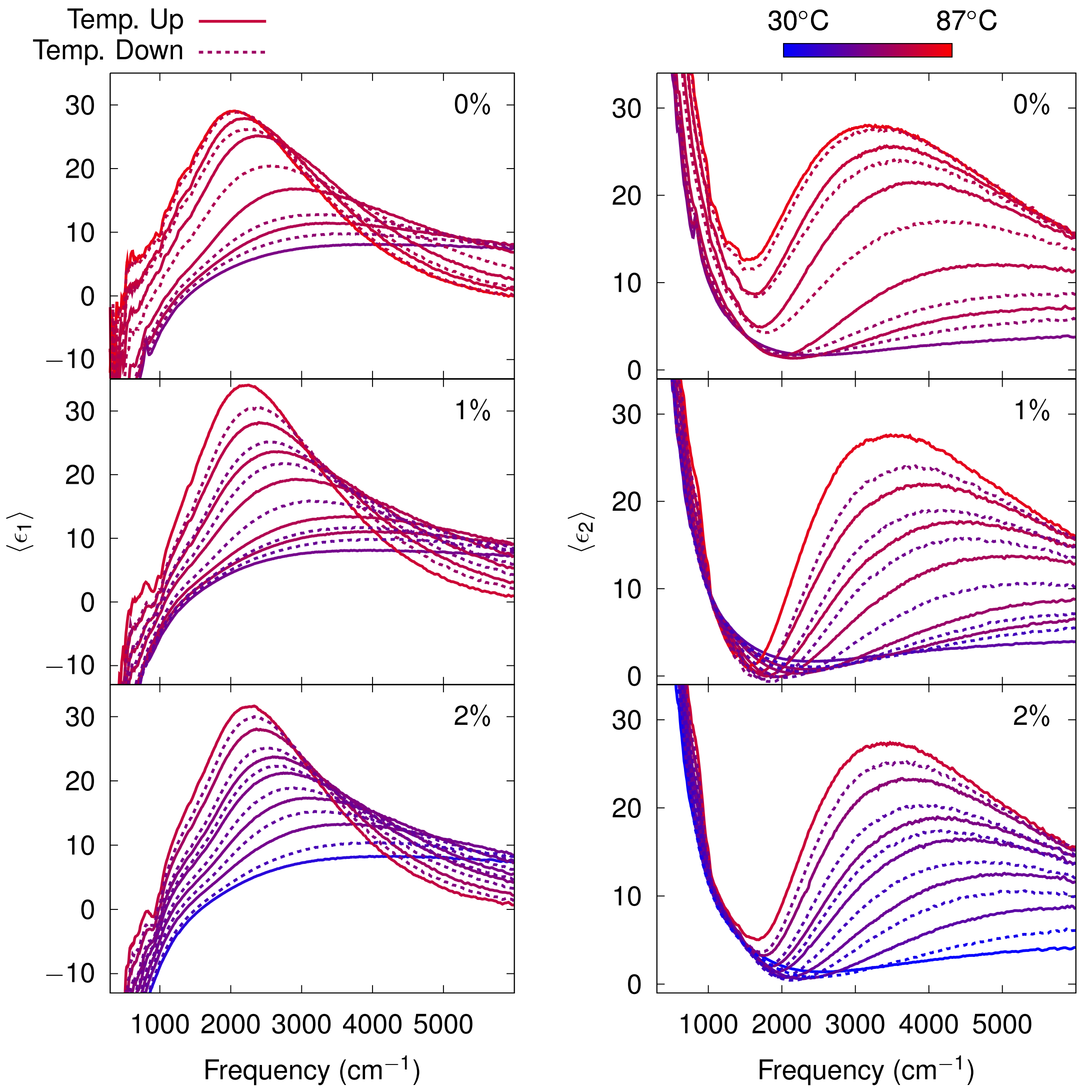

Fig. S2. Representative spectra of the real (left panels) and imaginary (right panels) parts of pseudo-dielectric function vs frequency, for different temperatures across the IMT. Solid and dashed lines correspond to rising and lowering temperatures, respectively. Corresponding doping percentages are indicated in each plot.

Pseudo-refractive index of silicon substrate. Figure S3 shows the real and imaginary parts of the pseudo-refractive index of the employed silicon substrate as a function of the frequency. The substrate owns a native silicon oxide layer of ~ 8 nm (as obtained from ellipsometric measurements). The plot displays a set of spectra from 18 to 115°C, which are observed to slightly change as a function of temperature (right insets), however, they do it with an appreciably lower shift as compared to those with VO_2 layers. Left insets show a close up to the Fano resonance associated to the highly doped Si substrate.

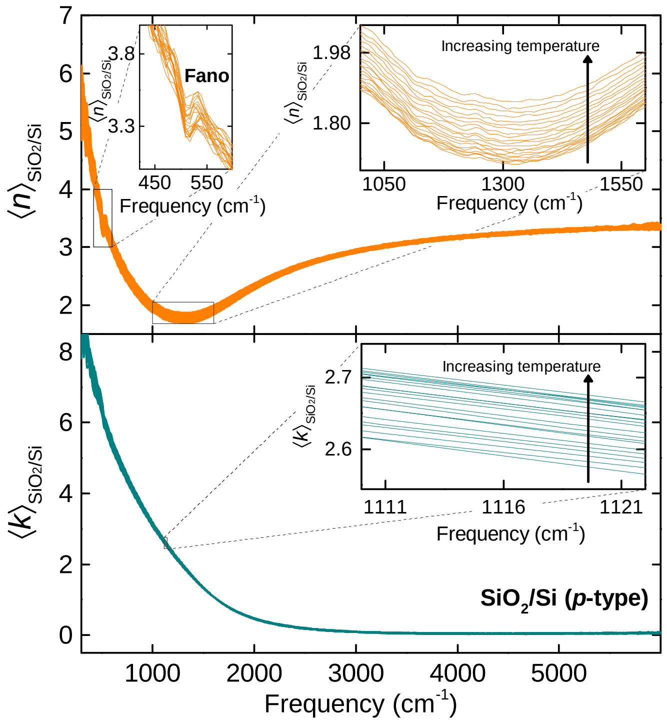

Fig. S3. Real $\langle n\rangle_{\mathrm{SiO_2/Si}}$ and imaginary $\langle k\rangle_{\mathrm{SiO_2/Si}}$ parts of pseudo-refractive index of silicon substrate, for different temperatures across the IMT. Left insets show a close up to a region of the Fano resonance for the highly doped bulk silicon. Right insets reveal the slight increments of spectral values with the temperature.

Hysteresis of integral pseudo-transforms

In Fig. 4(c) of the main text we compared successfully the integral of the optical conductivity for VO_2 with different Mo-doping concentrations to the corresponding growth laws as obtained from traces of the $\langle n\rangle$ representation at maximum. We provide here a further explanation for the usage of $\langle n\rangle$ to present data in Fig. 2 and the hysteresis of Fig. 3 the main paper. This is presented in Figure S4 in the form of comparison of integrals of $\langle n\rangle$ and of $\langle \epsilon_1\rangle$ against the result of Fig. 4(c).

It is observed that the integrals of $\langle n\rangle$ better represent the trends of integral conductivity, whereas the integrals of $\langle \epsilon_1\rangle$ always fail to the left. The reason for using real parts of both representations is that when dealing with overlayers there is an apparent interchange of real and imaginary parts (2, 3) that tends to be really marked when the overlayer has strong optical response as compared to the substrate (4). Actually, integrals for $\langle \epsilon_2\rangle$ were also made, but led to no fair comparison, as expected from the aforementioned interchange: they also form hysteresis but failed too much to the right. The reason for this lies in the fact, as our anonymous referees rightly pointed out the refractive index might peak at different position as the dielectric function when transforming one to the other, a situation that is clearly inherited to pseudo-transforms.

A further explanation for choosing $\langle n\rangle$ as a good representation of the observed physical phenomena should be given. In Fig. S5 hysteresis curves evaluated at the maximum of either $\langle n\rangle$ or $\langle \epsilon_1\rangle$ are presented. The point at which the traces are taken are shown at spectral positions with arrows in the left panel (their colors correspond to data presented in the other two panels), where direct comparisons of the real part of both pseudo-transforms are shown. Here it is seen that $\langle \epsilon_1\rangle$ is sharper around its maximum. The plot at the center depicts the hysteresis formed by traces at the frequency of the observed maximum of $\langle n\rangle$ for both pseudo transforms, obtaining quite similar line shapes. If, on the other hand, the trace is taken at the maximum of $\langle \epsilon_1\rangle$ (red symbols and lines in the rightmost panel), a hysteresis slightly but noticeably "delayed" is obtained instead. This indicates that thermally-freed carriers are resonating at the frequency of the apparent maximum of $\langle n\rangle$ even from the onset of the IMT. This is a relevant point considering that the maximum of $\langle \epsilon_1\rangle$ is not far from the maximum of the other pseudo-transform.

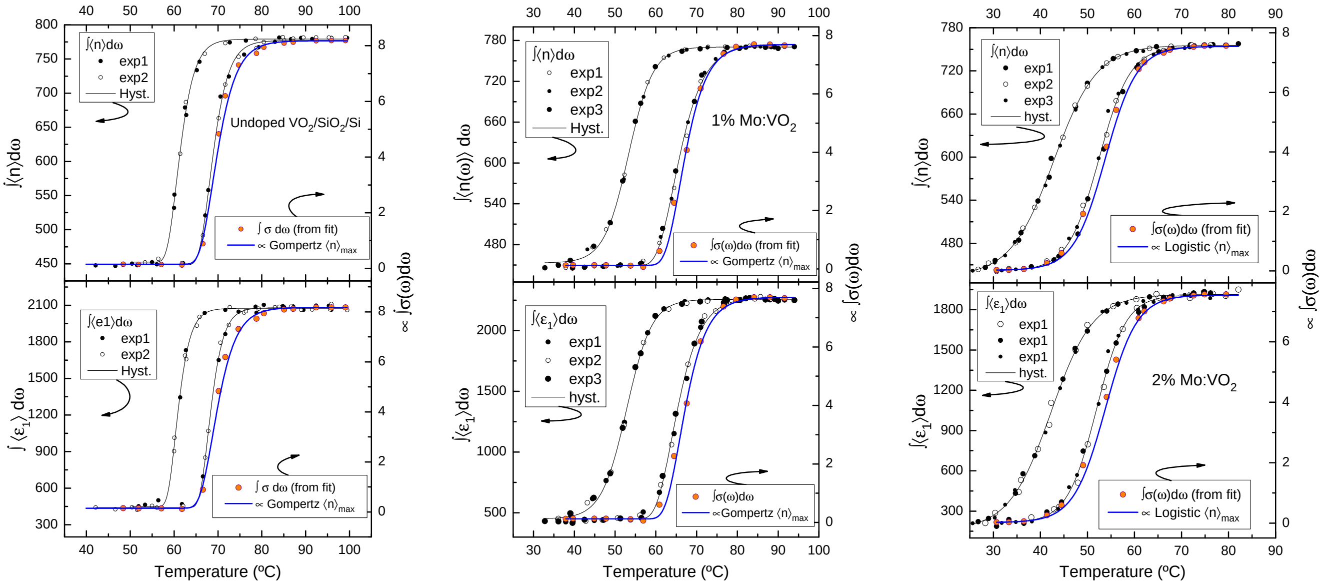

Fig. S4. Integrals of pseudo-transforms within the present working spectral range. Top panels: $\int \langle n \rangle \, d\omega$, bottom panels: $\int \langle \epsilon_1 \rangle \, d\omega$. (left to right) dopant concentration from 0 to 2% Mo. The black lines labeled as "hyst" are growth laws fits to the corresponding black symbols (i.e., to the integrals indicated in each frame)

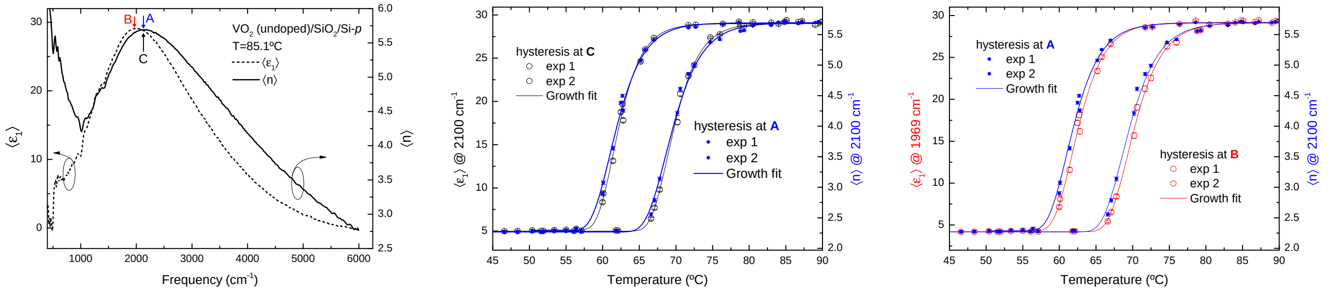

Fig. S5. (Left) Comparison of spectra of $\langle n \rangle$ and $\langle \epsilon_1 \rangle$ pseudo-transforms. (Center) Hysteresis of the indicated pseudo-transforms taken at the same frequency (i.e., that of $\langle n \rangle_{\max}$ indicated with A and C arrows in the left panel). (Right) Here the hysteresis of $\langle \epsilon_1 \rangle$ is taken at its maximum (corresponding to B-arrow in the left panel). When comparing with the hysteresis of $\langle n \rangle_{\max}$ a *delay* in temperature can be noted.

Spectral deconvolution example

The *deconvolution* of a selected experimental spectrum for pseudo-refractive index and pseudo-dielectric function by means of Drude-Smith, Fano and Drude-Smith+Fano models is shown in Figure S6.

Growth models

Gompertz model. The Gompertz growth law for the trace of $\langle n \rangle$ at maximum employed for ascending temperatures:

$$\langle n \rangle_{\max} = A_G \exp\left(-\exp\left(-k_G(T - T_G)\right)\right) + y_G, \qquad [2]$$

the values of A_G, k_G, T_G, and y_G for the different Mo concentrations are shown in table T1.

Logistic model. Logistic equation employed for both ascending and descending temperatures:

$$\langle n \rangle_{\max} = \frac{A_L}{1 + \exp\left(-k_L(T - T_L)\right)} + y_L, \qquad [3]$$

the values for A_L, k_L, T_L, and y_L are shown in table T1.

Dielectric function models

Dielectric function. The frequency-dependent general equation for the adjusted line shape of the dielectric function is

$$\tilde{\epsilon}(\omega) = \epsilon_1(\omega) + i\epsilon_2(\omega) \qquad [4]$$

$$= \tilde{\epsilon}_{DS}(\omega) + \tilde{\epsilon}_F(\omega) + \epsilon_\infty \qquad [5]$$

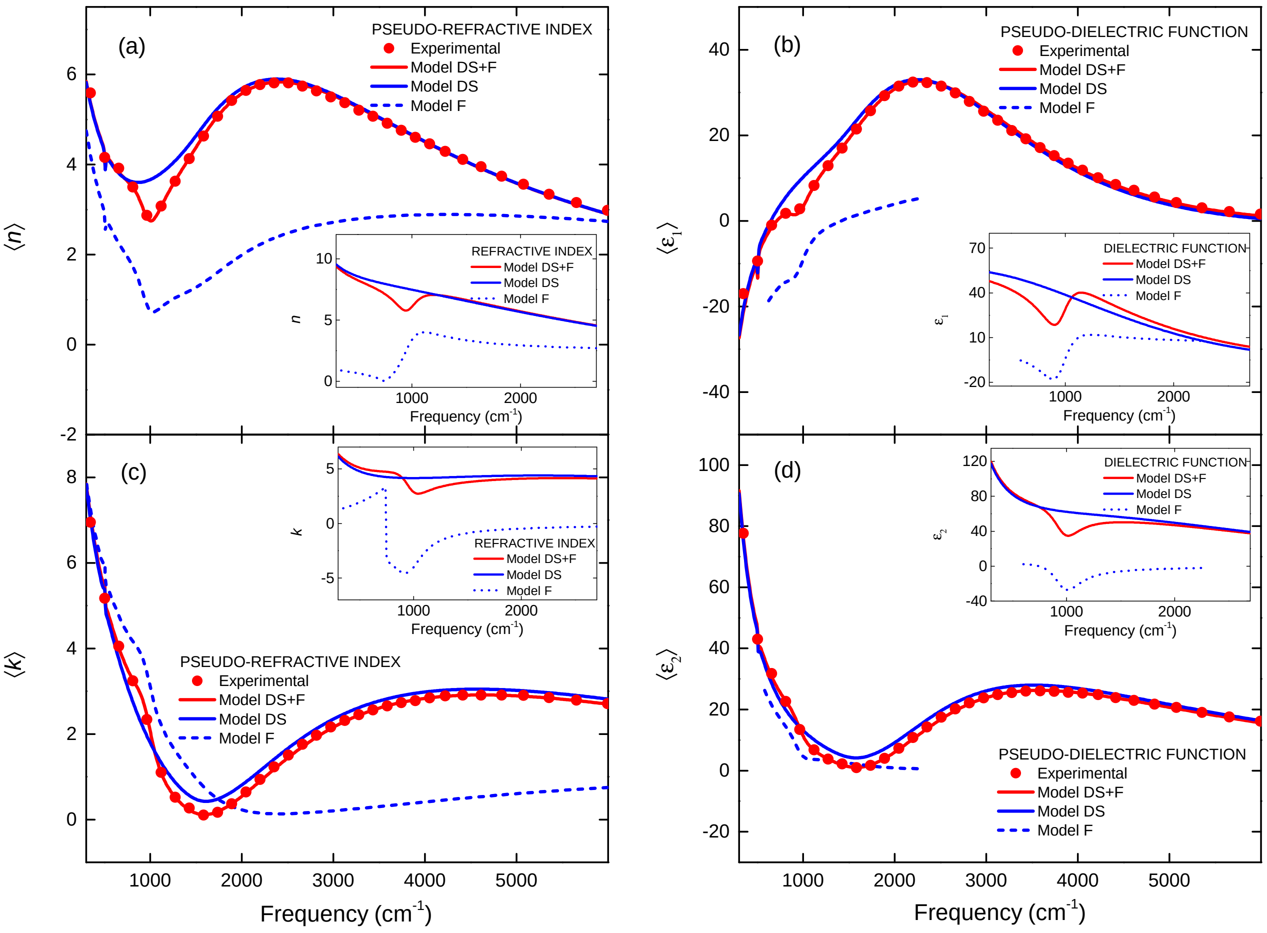

Fig. S6. (a) Real and (c) imaginary parts of pseudo refractive index of VO_2:Mo/Si as a function of the frequency for experimental data (red dots), and the fits through Drude-Smith with Fano model (red line). The $deconvolution$ in Drude-Smith and Fano single contributions are shown in blue lines as indicated in the panels. The inset shows n and k line shapes resulting from the adjustments of Drude-Smith+Fano (red line) along with the same $deconvolved$ curves (blue lines) as indicated. Panels (b) and (d) shows analogous information as (a) and (c) but for pseudo dielectric function.

Table T1. Parameters of Gompertz & logistic growth laws. The sub-index $\alpha = G, L$, depending on the growth law.

Mo doping	0 %		1 %		2 %	
Temp. direction	UP	DOWN	UP	DOWN	UP	DOWN
Growth law	Gompertz	Gompertz	Gompertz	Logistic	Logistic	Logistic
A_α	3.44605	3.43774	3.50687	3.50301	3.51612	3.56107
k_α	0.33848	0.38166	0.27907	0.3708	0.29717	0.23746
T_α	68.82605	61.05768	65.94226	54.3445	53.80033	44.32725
y_α	2.24192	2.25127	2.48193	2.47261	2.26668	2.22745

where $\epsilon_1(\omega)$ and $\epsilon_2(\omega)$ are the real and imaginary parts of complex function $\tilde{\epsilon}(\omega)$, respectively. $\tilde{\epsilon}_{DS}(\omega)$ and $\tilde{\epsilon}_F(\omega)$ correspond to the complex equations of Drude-Smith and Fano, respectively, and ϵ_∞ is the so called high-frequency permittivity limit.

The Drude-Smith model is described by

$$\mathrm{Re}\left[\tilde{\epsilon}_{DS}(\omega)\right] = -\omega_p^2\left[\frac{(1+2c)/\tau^2+\omega^2}{(1/\tau^2+\omega^2)^2}\right], \quad [6]$$

$$\mathrm{Im}\left[\tilde{\epsilon}_{DS}(\omega)\right] = \frac{\omega_p^2}{\omega\tau}\left[\frac{(1+c)/\tau^2+(1-c)\omega^2}{(1/\tau^2+\omega^2)^2}\right], \quad [7]$$

where

$$\omega_p^2 = \frac{Ne^2}{\epsilon_0 m^*}, \quad [8]$$

and ω_p, c, and τ, are the plasmon frequency, the persistency of velocity, and the collision-modified lifetime, respectively.

The Fano model is described by

$$\mathrm{Re}(\tilde{\epsilon}_F(\omega)) = A_F\left[\frac{2q+(1+q^2)(\hbar\omega-E_n)/\Gamma}{1+\left(\frac{\hbar\omega-E_n}{\Gamma}\right)^2}\right] \quad [9]$$

$$\mathrm{Im}(\tilde{\epsilon}_F(\omega)) = A_F\left[\frac{\left(q+\frac{\hbar\omega-E_n}{\Gamma}\right)^2}{1+\left(\frac{\hbar\omega-E_n}{\Gamma}\right)^2}\right] - A_F, \quad [10]$$

where A_F, q, Γ and E_n are the amplitude, the phase, the width of the resonant energy and the resonant energy, respectively. The fitting parameters are ω_p c, τ, A_F, q, E_n, Γ and ϵ_∞.

Optical conductivity. The real part of the optical conductivity in the Drude-Smith approach for a single scattering event, is (5)

$$\sigma_1(\omega) = \frac{Ne^2\tau/m^*}{1+\omega^2\tau^2}\left(1+\frac{c(1-\omega^2\tau^2)}{1+\omega^2\tau^2}\right) \quad [11]$$

where the second term in the RHS, weighed by the Drude denominator, is a function with the line shape of a single simple oscillation with equal weights above and below the ω axis.

Applying the f-sum rule:

$$\int_0^\infty \sigma_1(\omega)\,d\omega = \frac{Ne^2\tau}{m}\left(\frac{1}{\tau}\tan^{-1}(\omega\tau)+\frac{c\omega}{1+\omega^2\tau^2}\right)\Big|_0^\infty \quad [12]$$

The first term within the brackets yields the well-known f-sum rule result $\omega_p^2/8$ independent of measurement system, provided $\omega_p^2 = 4\pi Ne^2/m^*$ (cgs) and $Ne^2/(m^*\epsilon_0)$ (SI). The second term contributes 0 to the integral.

Fresnel coefficients

The interfacial reflection coefficients for polarizations of light perpendicular s and parallel p to the plane of incidence are (1)

$$r_{ij}^{(s)} = \frac{n_i\cos\theta_i - n_j\cos\theta_j}{n_i\cos\theta_i + n_j\cos\theta_j}, \quad [13]$$

$$r_{ij}^{(p)} = \frac{n_j\cos\theta_i - n_i\cos\theta_j}{n_j\cos\theta_i + n_i\cos\theta_j}, \quad [14]$$

where i and j are the media at both sides of the interface. Ellipsometry also considers the phase shift of the wave due its optical path though layer i, which is accounted for by

$$\beta_i = \frac{2\pi d_i}{\lambda}n_i\cos\theta_i. \quad [15]$$

The calculation of the total Fresnel coefficients for each polarization s or p is performed by calculating partial Fresnel coefficients in a cumulative way starting from the interface at the bottom of the stacked structure. Thus, if we label the media as follows: (0) = vacuum (or air), (1) = VO_2, (2) = SiO_2, and (3) = (semi-infinite) Si, then, the first Fresnel coefficients are simply the reflection coefficients $r_{23}^{(\alpha)}$ between SiO_2 and Si, where $\alpha = (s, p)$, the second coefficients are

$$r_{123}^{(\alpha)} = \frac{r_{12}^{\alpha} + r_{23}^{\alpha}\exp(-i2\beta_2)}{1 + r_{12}^{\alpha} r_{23}^{\alpha}\exp(-i2\beta_2)}, \qquad [16]$$

and finally the total Fresnel coefficients for the present system are

$$r_{0123}^{\alpha} = \frac{r_{01}^{\alpha} + r_{123}^{\alpha}\exp(-i2\beta_1)}{1 + r_{01}^{\alpha} r_{123}^{\alpha}\exp(-i2\beta_1)}. \qquad [17]$$

where the complex refractive index we are aiming to extract, i.e. $n_1 = n_{VO_2}(\omega, T)$ is inserted as argument of the reflection coefficients r_{01}, r_{12} and the phase shift β_1. The ellipsometric angles are then defined with the total Fresnel coefficients:

$$\Psi = \tan^{-1}\left|\frac{r_{0123}^{p}}{r_{0123}^{s}}\right| \equiv \tan^{-1}\left|\frac{r_p}{r_s}\right|, \qquad [18]$$

$$\Delta = \cos^{-1}\left(\arg[r_{0123}^{p}] - \arg[r_{0123}^{s}]\right) \equiv \cos^{-1}(\arg(r_p) - \arg(r_s)), \qquad [19]$$

where the equivalences at the right are the expressions used in Eq. (1) of the main text, for reasons of simplicity.

Temperature-dependent q-Fano parameter

Figure S7 shows the dependence of q-Fano parameter as a function of the temperature for VO_2 samples with the indicated Mo-doping percentage. It is noted that q, also called the shape factor of the Fano resonance, tends to zero (from below) when the IMT is completed independently of both, Mo-concentration and of the final morphology-conditioned state of conductivity of the film.

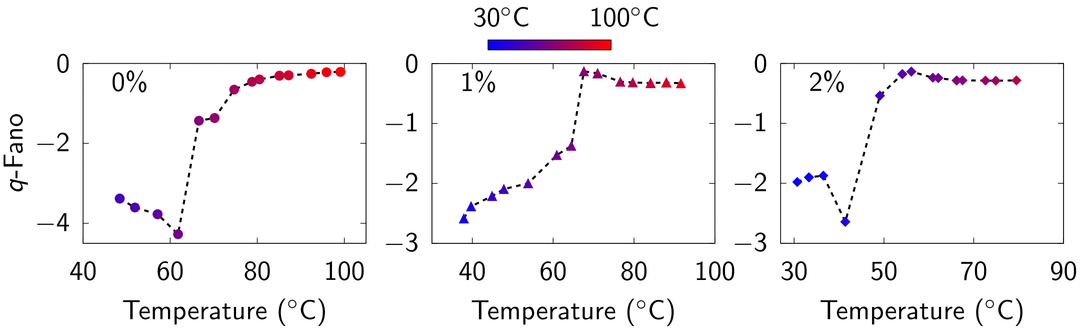

Fig. S7. q-Fano phase as a function of the temperature for the indicated percentages of Mo-doping.

References

1. H Fujiwara, *Spectroscopic Ellipsometry: Principles and Applications.* (John Wiley & Sons, Ltd), p. 82 (2007).
2. DE Aspnes, A Frova, Influence of spatially dependent perturbations on modulated reflectance and absorption of solids. *Solid State Commun.* **7**, 155–159 (1969).
3. MK Kelly, S Zollner, M Cardona, Modelling the optical response of surfaces measured by spectroscopic ellipsometry: application to Si and Ge. *Surf. Sci.* **285**, 282–204 (1993).
4. JM Flores-Camacho, et al., Optical anisotropies of metal clusters supported on a birefringent substrate. *Phys. Rev. B* **78**, 075416 (2008).
5. NV Smith, Classical generalization of the Drude formula for the optical conductivity. *Phys. Rev. B* **64**, 155106 (2001).
